# Supplementary material for: Polymodal K+ channel modulation contributes to dual analgesic and anti-inflammatory actions of traditional botanical medicines
Source: Commun Biol. 2024 Aug 28;7:1059. doi: 10.1038/s42003-024-06752-y (PMC11358443; doi:10.1038/s42003-024-06752-y)
Supplement: Supplementary file 2 — Supplmentary Information [file 42003_2024_6752_MOESM2_ESM.pdf]

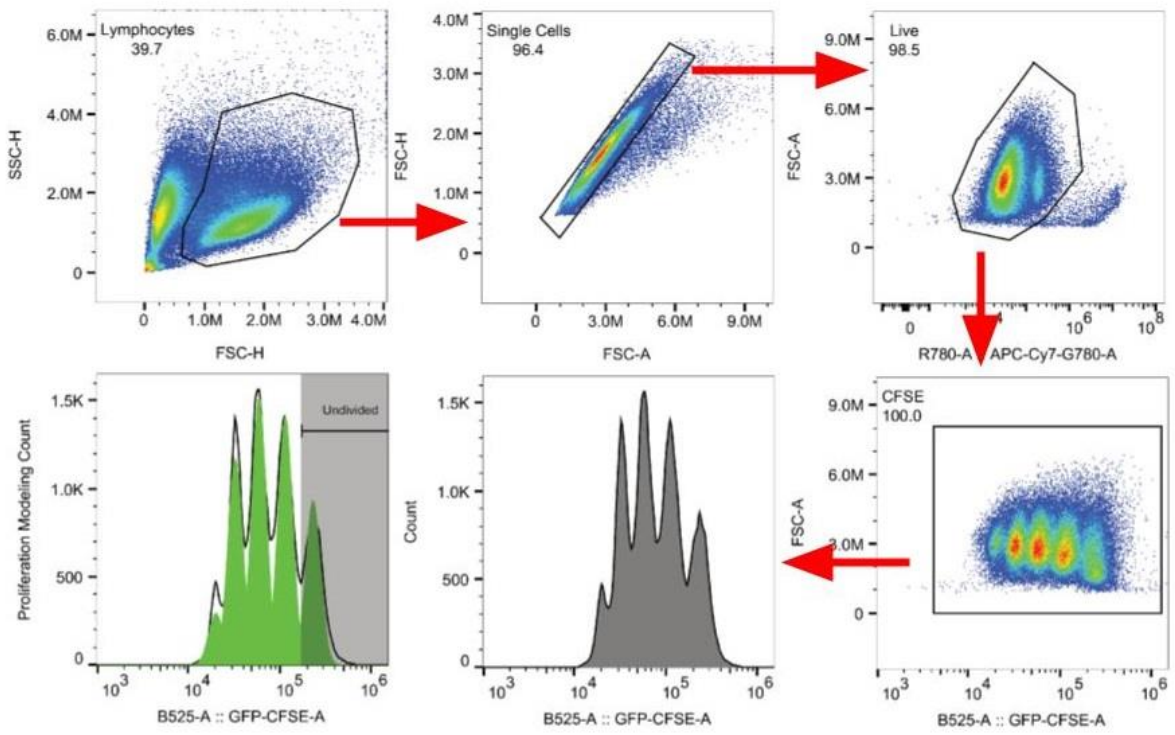


**Supplementary Figure 1.** Gating strategy and proliferation modeling: Sequential gating strategy to identify lymphocytes, exclude doublets, define live cells, and model CFSE dye dilution to estimate division index.

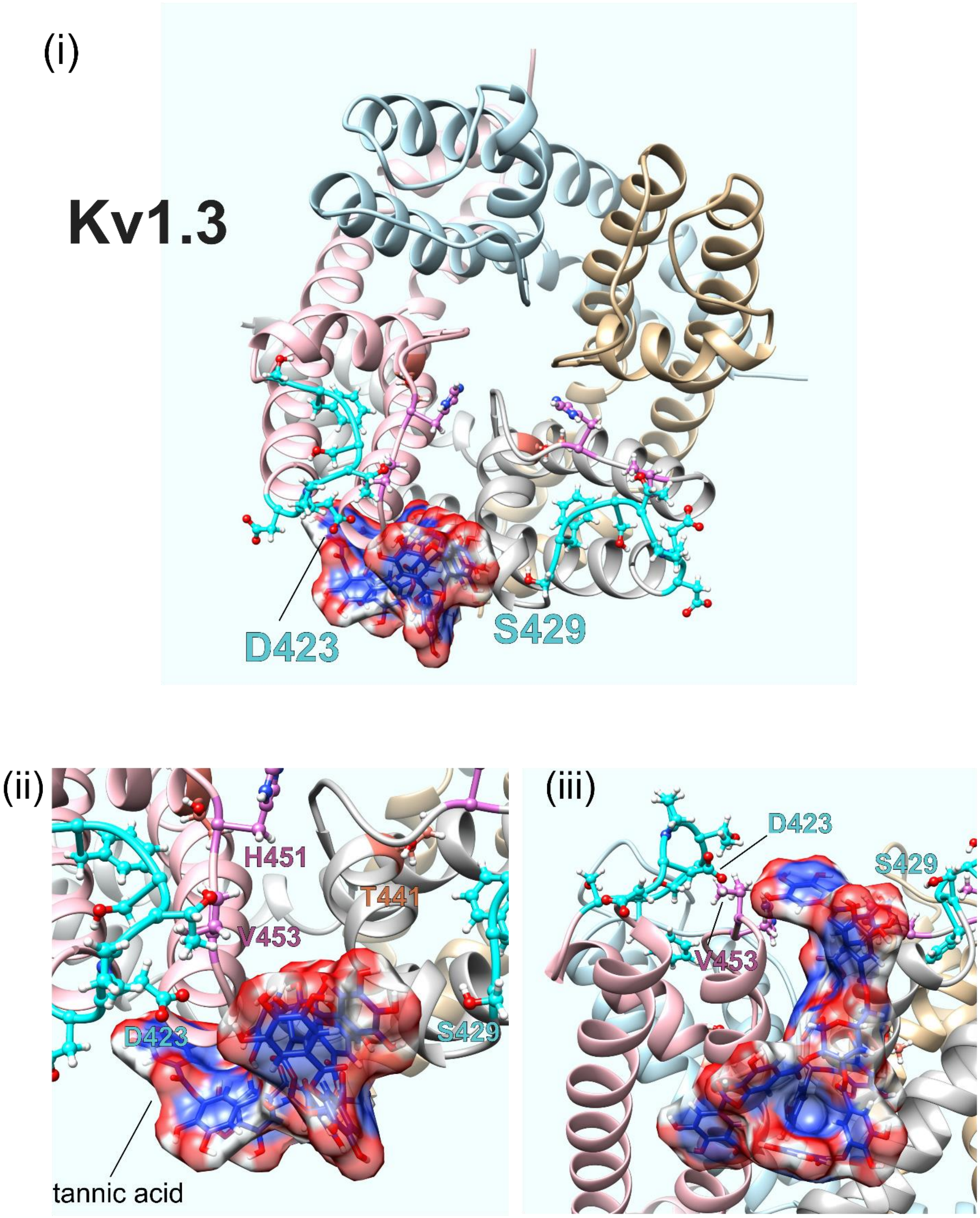

(i)
Kv1.3
D423
S429
(ii)
H451
T441
V453
D423
S429
tannic acid
(iii)
D423
S429
V453

**Supplementary Figure 2. Enlarged view of Figure 8C**.

Kv1.3 pore module structure with tannic acid (red, white and blue) docked using SwissDock; divergent residues from two adjoining α subunits are colored as in Figure 8A. (i) top view; (ii) top view closeup; (iii) side view.

**Supplementary Table 1. Compounds Identified in Fireweed Extract by MS and MS/MS analysis[x]**

| Compound | Parent mass (m/z) | Daughter ion(s) (m/z) |
|---|---|---|
| Gallic acid | 169 | 125 |
| Quinic acid | 191 | 173, 171 |
| Ellagic acid | 301 | ND |
| (Iso)chlorogenic acid | 353 | 191, 179, 173 |
| Quercitrin | 447 | ND |
| Hyperoside | 463 | ND |
| Hyperin gallate | 615 | ND |
| Oenothein B | 783[x] | ND |

[x]Fireweed extract was diluted 100-fold in MeOH and directly injected on MS

[y]Oenothein (MW 1569) shows $[M-2H]^{-2}$

ND is not determined

**Supplementary Table 2. Compounds Identified in Witch Hazel Extract by MS and MS/MS analysis[x]**

| Compound | Parent mass (m/z) | Daughter ion(s) (m/z) |
|---|---|---|
| Gallic acid | 169 | 125 |
| Glucose monogallate | 331 | ND |
| Glucose digallate | 483, 967[y] | 313, 241, 169 |
| Glucose trigallate | 635 | 483, 313, 223, 169 |

[x]Witch hazel extract was diluted 100-fold in MeOH and directly injected on MS

[y]Glucose digallate (or isomer including hamamelitannin) shows $M-H^+$ and $2M-H^+$

| Kv7.2/7.3 | $V_{0.5}$ non-normalized tail current (mV) | Slope (mV) |
|---|---|---|
| Control | -40.78 ± 1.12 (n=10) | 6.39 ± 0.55 (n=10) |
| 1:100 Fireweed Extract | n.a. (n=10) | n.a. (n=10) |

**Supplementary Table 3.** Kv7.2/7.3 in absence or presence of fireweed. Values indicate mean ± SEM.

| Kv7.2/7.3 | Tau (ms) |
|---|---|
| -40 mV | 740.61 ± 57.19 (*n*=6) |
| -20 mV | 490.22 ± 78.67 (*n*=10) |
| 0 mV | 335.46 ± 66.34 (*n*=9) |
| 20 mV | 169.76 ± 21.74 (*n*=8) |
| 40 mV | 103.81 ± 7.27 (*n*=8) |

**Supplementary Table 4.** Kv7.2/7.3 in 4 mM $[K^+]_O$. Values indicate mean ± SEM.

| Kv7.2/7.3 | Tau Fast (ms) | Tau Slow (ms) |
|---|---|---|
| -20 mV | 319.63 ± 16.97 (*n*=9) | 3170.82 ± 271.21 (*n*=9) |
| 0 mV | 209.74 ± 11.58 (*n*=9) | 2325.44 ± 194.50 (*n*=9) |
| 20 mV | 185.51 ± 11.94 (*n*=9) | 2143.81 ± 212.71 (*n*=9) |
| 40 mV | 180.59 ± 12.76 (*n*=9) | 1686.24 ± 194.42 (*n*=9) |

**Supplementary Table 5.** Kv7.2/7.3 in the presence of fireweed. Values indicate mean ± SEM.

| Kv1.3 | Peak current at +40 (mV) |
|---|---|
| Control | 7.63 ± 0.84 (n=12) |
| 1:100 Fireweed Extract | 4.55 ± 0.50 ($p<0.0001$; *n*=12) |

**Supplementary Table 6.** Statistics versus Kv1.3 in absence of fireweed. Values indicate mean ± SEM. Statistical comparisons by paired t-test.

| Kv1.3 | Tau (ms) |
|---|---|
| -30 mV | 14.18 ± 1.21 (*n*=12) |
| -20 mV | 7.87 ± 0.84 (*n*=12) |
| -10 mV | 5.16 ± 0.56 (*n*=12) |
| 0 mV | 3.99 ± 0.42 (*n*=12) |
| 10 mV | 3.42 ± 0.36 (*n*=12) |
| 20 mV | 3.11 ± 0.34 (*n*=12) |
| 30 mV | 2.83 ± 0.32 (*n*=12) |
| 40 mV | 2.75 ± 0.33 (*n*=12) |

**Supplementary Table 7.** Kv1.3 in 4 mM $[K^+]_O$. Values indicate mean ± SEM.

| Kv1.3 | Tau Fast (ms) | Tau Slow (ms) |
|---|---|---|
| -30 mV | 55.08 ± 3.39 (*n*=11) | 247.28 ± 20.93 (*n*=11) |
| -20 mV | 54.57 ± 4.52 (*n*=12) | 245.15 ± 21.90 (*n*=12) |
| -10 mV | 51.51 ± 4.87 (*n*=12) | 236.60 ± 25.19 (*n*=12) |
| 0 mV | 46.69 ± 4.79 (*n*=12) | 227.08 ± 24.72 (*n*=12) |
| 10 mV | 41.71 ± 5.47 (*n*=12) | 213.73 ± 22.70 (*n*=12) |
| 20 mV | 37.88 ± 4.66 (*n*=12) | 198.84 ± 21.42 (*n*=12) |
| 30 mV | 34.38 ± 4.23 (*n*=12) | 179.97 ± 19.38 (*n*=12) |
| 40 mV | 31.46 ± 3.83 (*n*=12) | 161.48 ± 16.38 (*n*=12) |

**Supplementary Table 8.** Kv1.3 in the presence of fireweed. Values indicate mean ± SEM.

| **Kv1.3** | **Current at 0 (mV)** | **$V_{0.5}$ Normalized tail current (mV)** | **Slope (mV)** |
|---|---|---|---|
| Control | 1.99 ± 0.28 (*n*=5) | -27.75 ± 1.03 (*n*=5) | 4.86 ± 0.31 (*n*=5) |
| 0.1 µM | 2.39 ± 0.41 (*p*=0.3718; *n*=5) | -27.80 ± 2.36 (*p*>0.9999; *n*=5) | 4.70 ± 0.45 (*p*>0.9999; *n*=5) |
| 1 µM | 2.23 ± 0.35 (*p*=0.5066; *n*=5) | -28.17 ± 3.08 (*p*>0.9999; *n*=5) | 4.73 ± 0.52 (*p*>0.9999; *n*=5) |
| 3 µM | 1.96 ± 0.31 (*p*=0.9999; *n*=5) | -28.73 ± 3.31 (*p*>0.9999; *n*=5) | 5.21 ± 0.43 (*p*>0.9998; *n*=5) |
| 10 µM | 1.09 ± 0.25 (*p*=0.0472; *n*=5) | -25.49 ± 4.06 (*p*=0.9904; *n*=5) | 7.44 ± 0.95 (*p*=0.3488; *n*=5) |
| 30 µM | 0.72 ± 0.27 (*p*=0.0380; *n*=4) | -19.06 ± 4.68 (*p*=0.2903; *n*=4) | 10.91 ± 2.27 (*p*=0.0038; *n*=4) |
| 100 µM | 0.32 ± 0.08 (*p*=0.0170; *n*=4) | -9.91 ± 3.45 (*p*=0.0042; *n*=4) | 15.18 ± 2.02 (<0.0001; *n*=4) |

**Supplementary Table 9.** Statistics versus Kv1.3 in absence of tannic acid. Values indicate mean ± SEM. Statistical comparisons by One-Way ANOVA, with Dunnett's multiple comparison test.

| **Kv7.2/7.3** | **$V_{0.5}$ non-normalized tail current (mV)** | **Slope (mV)** |
|---|---|---|
| Control | -40.85 ± 1.79 (n=11) | 7.32 ± 0.21 (n=11) |
| 1:100 Witch Hazel Bark Extract | -51.96 ± 2.89 (*p<0.0001*; *n*=11) | 12.178 ± 0.84 (*p<0.0001*; *n*=11) |

**Supplementary Table 10.** Statistics versus Kv1.3 in absence of witch hazel bark. Values indicate mean ± SEM. Statistical comparisons by paired t-test.

| Kv7.5 | $V_{0.5}$ non-normalized tail current (mV) | $V_{0.5}$ Normalized tail current (mV) | Slope (mV) |
|---|---|---|---|
| Control | -40.11 ± 1.14 (*n*=6) | -39.56 ± 1.05 (*n*=6) | 9.13 ± 1.29 (*n*=6) |
| 1:100 Witch Hazel Bark Extract | -78.75 ± 9.68 (*p*=0.0075; *n*=6) | -78.59 ± 9.1 (*p*=0.0078; *n*=6) | 19.97 ± 1.65 (*p*=0.0035; *n*=6) |

**Supplementary Table 11.** Statistics versus Kv7.5 in absence of witch hazel bark. Values indicate mean ± SEM. Statistical comparisons by paired t-test.

| Kv7.5 | $V_{0.5}$ non-normalized tail current (mV) | $V_{0.5}$ Normalized tail current (mV) | Slope (mV) |
|---|---|---|---|
| Control | -50.27 ± 1.37 (*n*=4) | -48.15 ± 1.14 (*n*=4) | 7.66 ± 0.33 (*n*=4) |
| 100 µM Hamamelitannin | -49.87 ± 1.44 (*p*=0.2524; *n*=4) | -47.96 ± 0.99 (*p*=0.7507; *n*=4) | 7.57 ± 0.17 (*p*=0.7092; *n*=4) |

**Supplementary Table 12.** Statistics versus Kv7.5 in absence of hamamelitannin. Values indicate mean ± SEM. Statistical comparisons by paired t-test.

| Kv1.3 | Peak current at +40 (mV) | $V_{0.5}$ Normalized tail current (mV) | Slope (mV) |
|---|---|---|---|
| Control | 0.23 ± 0.03 (*n*=6) | -13.30 ± 1.45 (*n*=6) | 6.52 ± 0.86 (*n*=6) |
| 1:100 Witch Hazel Bark Extract | 0.04 ± 0.01 (*p*=0.0009; *n*=6) | n.a. | n.a. |

**Supplementary Table 13.** Statistics versus Kv1.3 in absence of witch hazel bark. Values indicate mean ± SEM. Statistical comparisons by paired t-test.

| Kv1.1 | $V_{0.5}$ non-normalized tail current (mV) | $V_{0.5}$ Normalized tail current (mV) | Slope (mV) |
|---|---|---|---|
| Control | -31.99 ± 2.35 (*n*=8) | -30.51 ± 2.37 (*n*=8) | 6.75 ± 0.35 (*n*=8) |
| 1:100 Witch Hazel Bark Extract | -32.19 ± 2.40 (*p*=0.9042; *n*=8) | -31.35 ± 2.34 (*p*=0.6401; *n*=8) | 8.07 ± 0.45 (*p*=0.0628; *n*=8) |

**Supplementary Table 14.** Statistics versus Kv1.1 in absence of witch hazel bark. Values indicate mean ± SEM. Statistical comparisons by paired t-test.

| Kv1.1 | $V_{0.5}$ non-normalized tail current (mV) | $V_{0.5}$ Normalized tail current (mV) | Slope (mV) |
|---|---|---|---|
| Control | -35.49 ± 1.26 (*n*=8) | -33.71 ± 1.43 (*n*=8) | 5.80 ± 0.33 (*n*=8) |
| 100 µM Catechin hydrate | -39.60 ± 0.63 (*p*=0.0141; *n*=8) | -37.70 ± 0.45 (*p*=0.0225; *n*=8) | 6.10 ± 0.15 (*p*=0.2044; *n*=8) |

**Supplementary Table 15.** Statistics versus Kv1.1 in absence of catechin hydrate. Values indicate mean ± SEM. Statistical comparisons by paired t-test.

| Kv1.1 | $V_{0.5}$ non-normalized tail current (mV) | $V_{0.5}$ Normalized tail current (mV) | Slope (mV) |
|---|---|---|---|
| Control | -28.74 ± 2.20 (*n*=8) | -27.97 ± 2.12 (*n*=8) | 6.55 ± 0.66 (*n*=8) |
| 100 µM Hamamelitannin | -36.25 ± 1.77 (*p*=0.0019; *n*=8) | -35.11 ± 1.66 (*p*=0.020; *n*=8) | 6.51 ± 0.73 (*p*=0.8617; *n*=8) |

**Supplementary Table 16.** Statistics versus Kv1.1 in absence of hamamelitannin. Values indicate mean ± SEM. Statistical comparisons by paired t-test.

| TREK-1 | Peak current at +40 (mV) |
|---|---|
| Control | 0.70 ± 0.08 (*n*=9) |
| 1:100 Witch Hazel Bark Extract | 3.72 ± 0.43 (<0.0001; *n*=9) |

**Supplementary Table 17.** Statistics versus TREK-1 in absence of witch hazel bark. Values indicate mean ± SEM. Statistical comparisons by paired t-test.

| TREK-1 | Current at 0 (mV) |
|---|---|
| Control | 0.23 ± 0.03 (*n*=5) |
| 10 µM | 0.44 ± 0.08 (*p*=0.7665; *n*=5) |
| 50 µM | 0.89 ± 0.11 (*p*=0.0197; *n*=5) |
| 100 µM | 1.37 ± 0.14 (<0.0001; *n*=5) |
| 250 µM | 1.86 ± 0.19 (<0.0001; *n*=5) |
| 500 µM | 2.22 ± 0.24 (<0.0001; *n*=5) |

**Supplementary Table 18.** Statistics versus TREK-1 in absence of tannic acid. Values indicate mean ± SEM. Statistical comparisons by One-Way ANOVA, with Dunnett's multiple comparison test.

| Kv1.3 Mutant 1 | Current at 0 (mV) | $V_{0.5}$ Normalized tail current (mV) | Slope (mV) |
|---|---|---|---|
| Control | 4.66 ± 1.27 (*n*=4) | -14.75 ± 3.06 (*n*=4) | 3.97 ± 0.40 (*n*=4) |
| 0.1 µM | 5.24 ± 0.91 (*p*=0.9779; *n*=4) | -20.05 ± 2.13 (*p*=0.6097; *n*=4) | 5.16 ± 0.56 (*p*=0.1982; *n*=4) |
| 1 µM | 5.37 ± 0.63 (*p*=0.9475; *n*=4) | -24.05 ± 2.18 (*p*=0.1294; *n*=4) | 4.97 ± 0.53 (*p*=0.3410; *n*=4) |
| 3 µM | 4.93 ± 0.57 (*p*=0.9996; *n*=4) | -27.01 ± 1.83 (*p*=0.0287; *n*=4) | 5.05 ± 0.28 (*p*=0.2776; *n*=4) |
| 10 µM | 4.01 ± 0.41 (*p*=0.9660; *n*=4) | -27.63 ± 1.98 (*p*=0.0205; *n*=4) | 5.33 ± 0.20 (*p*=0.1123; *n*=4) |
| 30 µM | 2.18 ± 0.24 (*p*=0.0896; *n*=4) | -24.82 ± 2.70 (*p*=0.0896; *n*=4) | 5.94 ± 0.30 (*p*=0.0117; *n*=4) |
| 100 µM | 1.15 ± 0.14 (*p*=0.0095; *n*=4) | -19.79 ± 4.83 (*p*=0.6542; *n*=4) | 6.41 ± 0.42 (*p*=0.0018; *n*=4) |

**Supplementary Table 19.** Statistics versus Kv1.3 Mutant 1 in absence of tannic acid. Values indicate mean ± SEM. Statistical comparisons by One-Way ANOVA, with Dunnett's multiple comparison test.

| **Kv1.3 Mutant 3** | **Current at 0 (mV)** | **$V_{0.5}$ Normalized tail current (mV)** | **Slope (mV)** |
|---|---|---|---|
| Control | 3.39 ± 0.48 (*n*=4) | -31.00 ± 0.53 (*n*=4) | 2.50 ± 0.70 (*n*=4) |
| 0.1 µM | 4.20 ± 0.53 (*p*=0.6634; *n*=4) | -30.20 ± 0.78 (*p*=0.9995; *n*=4) | 4.01 ± 0.35 (*p*=0.5998; *n*=4) |
| 1 µM | 4.44 ± 0.64 (*p*=0.4195; *n*=4) | -30.51 ± 0.97 (>0.9999; *n*=4) | 3.67 ± 0.41 (*p*=0.7897; *n*=4) |
| 3 µM | 4.25 ± 0.64 (*p*=0.6081; *n*=4) | -30.85 ± 1.34 (>0.9999; *n*=4) | 4.85 ± 0.24 (*p*=0.2211; *n*=4) |
| 10 µM | 2.28 ± 0.38 (*p*=0.3671; *n*=4) | -29.05 ± 1.94 (*p*=0.9703; *n*=4) | 6.40 ± 0.62 (*p*=0.0179; *n*=4) |
| 30 µM | 0.88 ± 0.13 (*p*=0.0050; *n*=4) | -16.81 ± 5.40 (*p*=0.0032; *n*=4) | 14.11 ± 1.75 (<0.0001; *n*=4) |
| 100 µM | 0.50 ± 0.07 (*p*=0.0013; *n*=4) | n.a. | n.a. |

**Supplementary Table 20.** Statistics versus Kv1.3 Mutant 1 in absence of tannic acid. Values indicate mean ± SEM. Statistical comparisons by One-Way ANOVA, with Dunnett's multiple comparison test.

| Donor | Sex | Age | Race/ Ethnicity |
|---|---|---|---|
| Donor 1 | Male | 54 | White/ Non-Hispanic |
| Donor 2 | Female | 51 | Asian/ Non-Hispanic |
| Donor 3 | Male | 37 | White/ Hispanic |
| Donor 4 | Female | 49 | White/ Asian |
| Donor 5 | Male | 26 | Other/ Non-Hispanic |
| Donor 6 | Female | 50 | Asian/ Non-Hispanic |
| Donor 7 | Male | 56 | White/ Non-Hispanic |
| Donor 8 | Male | 75 | White/ Non-Hispanic |
| Donor 9 | Female | 40 | White/ Hispanic |
| | | | |

**Supplementary Table 21. Gender, Age and Ethnicity of PMBC donors for this study**

| **cluster** | **ΔG (kcalmol$^{-1}$)** |
|---|---|
| 25 | -11.94 |
| **11** | **-11.31** |
| 23 | -11.16 |
| 9 | -11.14 |
| 0 | -11.02 |
| 14 | -10.98 |
| 30 | -10.84 |
| 3 | -10.70 |
| 7 | -10.54 |
| 16 | -10.28 |

**Supplementary Table 22.** The top 10 clusters of binding poses for tannic acid to Kv1.3 ranked by ΔG free energy (see Figure 7C). Bold – pose shown in Figure 7C.

| **cluster** | **ΔG (kcalmol$^{-1}$)** |
|---|---|
| 21 | -11.12 |
| 11 | -10.98 |
| **3** | **-10.91** |
| 17 | -10.80 |
| 29 | -10.74 |
| 9 | -10.70 |
| 16 | -10.65 |
| 27 | -10.38 |
| 28 | -10.13 |
| 14 | -10.11 |

**Supplementary Table 23.** The top 10 clusters of binding poses for tannic acid to Kv1.2 ranked by ΔG free energy (see Figure 7D). Bold – pose shown in Figure 7D.
